# Individual periovulatory ovarian responses associated with pregnancy outcome following an Ovsynch protocol in Peranakan Ongole cows

**DOI:** 10.3389/fvets.2026.1920158

**Published:** 2026-09-14

**Authors:** Sari Yanti Hayanti, Muhammad Imron, Santoso Santoso, Ahmad Adigunawan, Rahma Isartina Anwar, Adelaide José Pereira Cristóvão, Mohamad Agus Setiadi, Herdis Herdis, Bambang Purwantara, Mokhamad Fakhrul Ulum, Amrozi Amrozi

**Affiliations:** 1 Study Program of Veterinary Biomedical Sciences, Postgraduate School, IPB University, Bogor, West Java, Indonesia; 2 Research Center for Animal Husbandry, Research Organization of Agriculture and Food, National Research and Innovation Agency (BRIN), Bogor, Indonesia; 3 Center for Livestock Breeding and Forage Feed Development, Ministry of Agriculture, Sembawa, South Sumatra, Indonesia; 4 Animal Health Department, Faculty of Agriculture, Universidade Nacional Timor Lorosa’e (UNTL), Dili, Timor-Leste; 5 Division of Reproduction and Obstetrics, School of Veterinary Medicine and Biomedical Sciences, IPB University, Bogor, West Java, Indonesia

**Keywords:** *Bos indicus*, corpus hemorrhagicum, luteinization, ovarian dynamics, ovulation synchronization, periovulatory, reproductive efficiency, ultrasonography

## Abstract

**Introduction:**

Although ovulation synchronization protocols effectively control the timing of ovulation, pregnancy success remains highly variable among individual cows. The periovulatory ovarian responses associated with subsequent pregnancy outcome following synchronization remain poorly characterized in *Bos indicus* beef cattle. Therefore, this exploratory longitudinal study characterized individual ovarian sonographic and hormonal responses during an Ovsynch synchronization protocol and evaluated associations with pregnancy outcome in Peranakan Ongole cows.

**Methods:**

Ten multiparous Peranakan Ongole cows without apparent reproductive abnormalities and body condition scores of 2.5–3.0 (5-point scale) were synchronized using a single Ovsynch protocol. Ovarian sonographic characteristics and circulating estradiol and progesterone concentrations were evaluated throughout the periovulatory period, and pregnancy was determined by ultrasonography.

**Results:**

Nine of the ten cows ovulated following the synchronization protocol, and pregnancy was confirmed in four cows. Most post-ovulatory ovarian and hormonal variables showed weak associations at the 72-h post-ovulatory assessment. Baseline ovarian characteristics showed no detectable associations with post-ovulatory responses, whereas pre- and post-ovulatory estradiol concentrations remained positively associated. Among the post-ovulatory variables evaluated, corpus hemorrhagicum diameter showed the strongest nominal association with pregnancy outcome. Combining corpus hemorrhagicum diameter with largest follicle diameter or corpus hemorrhagicum echogenicity also showed nominal associations.

**Conclusion:**

These preliminary findings suggest that early post-ovulatory ovarian characteristics, particularly corpus hemorrhagicum development, may be relevant to pregnancy outcome following synchronization.

## 1 Introduction

Achieving one calf per year per cow is a crucial objective in large-scale cattle farming, particularly for beef production systems. Efforts to attain this target are often hindered by inefficiencies in estrus detection (1). The GnRH-PGF2α-GnRH Ovsynch protocol was developed to address this inefficiency by enabling scheduled artificial insemination (AI) without reliance on estrus detection (2, 3). However, even under standardized protocols, pregnancy rates remain variable (4, 5). This variability is particularly pronounced in tropical beef production systems, where additional challenges such as heat stress, nutritional constraints, and the predominance of *Bos indicus* cattle may influence protocol efficacy.

Several studies have examined factors contributing to this variability. Abdallah et al. (6) demonstrated that cows receiving the Ovsynch protocol achieved ovulation success rates of up to 100%, yet ovulation occurred on different days among individuals. This variability in ovulation timing is thought to be influenced by differences in pre-ovulatory follicle morphology (7) and the ovulatory response to the first GnRH administration (8). Other studies have evaluated progesterone and estradiol profiles (9) and post-ovulatory morphological and functional characteristics in relation to pregnancy (10). Although numerous studies have investigated ovarian morphology and endocrine responses following Ovsynch, the components have largely been evaluated independently. Consequently, the coordinated ovarian response throughout the periovulatory period and its relationship with pregnancy establishment remain poorly understood. This persistent variability suggests that additional, unexplored physiological factors, such as the relationship between pre-ovulatory and post-ovulatory ovarian structure, may contribute to pregnancy outcomes.

Pregnancy outcome depends not only on successful ovulation but also on coordinated structural and endocrine changes throughout the periovulatory period (11). Therefore, this exploratory study aimed to characterize individual ovarian sonographic and hormonal responses throughout the Ovsynch-based synchronization protocol in Peranakan Ongole cows and to evaluate their relationships with early pregnancy outcome.

## 2 Materials and methods

### 2.1 Research time and location

The research was conducted from May 2024 to February 2025 at the Center for Livestock Breeding and Forage Feed Development (BPTU and HPT) in Sembawa, South Sumatra, Indonesia and the Agro-Biomedical Industrial Technology Development Laboratory (LAPTIAB) BRIN in Serpong, Banten, Indonesia.

### 2.2 Animal selection

The study utilized Peranakan Ongole cows. From an initial pool of 472 cows aged 48–120 months, transrectal ultrasonography identified 59 non-pregnant cows without apparent reproductive abnormalities. Further screening for a body condition score of 2.5–3 (scale 1–5) narrowed the pool to 20 multiparous cows (≥2 parities). Reproductive records were reviewed as supporting information during animal selection. Final classification of cows into the mid- to late luteal phase was based on transrectal B-mode ultrasonographic evaluation of ovarian morphology according to the criteria described by Kojima (12). These 10 cows (coded a˗j) received the Ovsynch-based synchronization protocol. All animals were maintained on a consistent diet (10% BW feed/fodder) with water provided ad libitum. The selection cascade is illustrated in Figure 1A.

**Figure 1:**
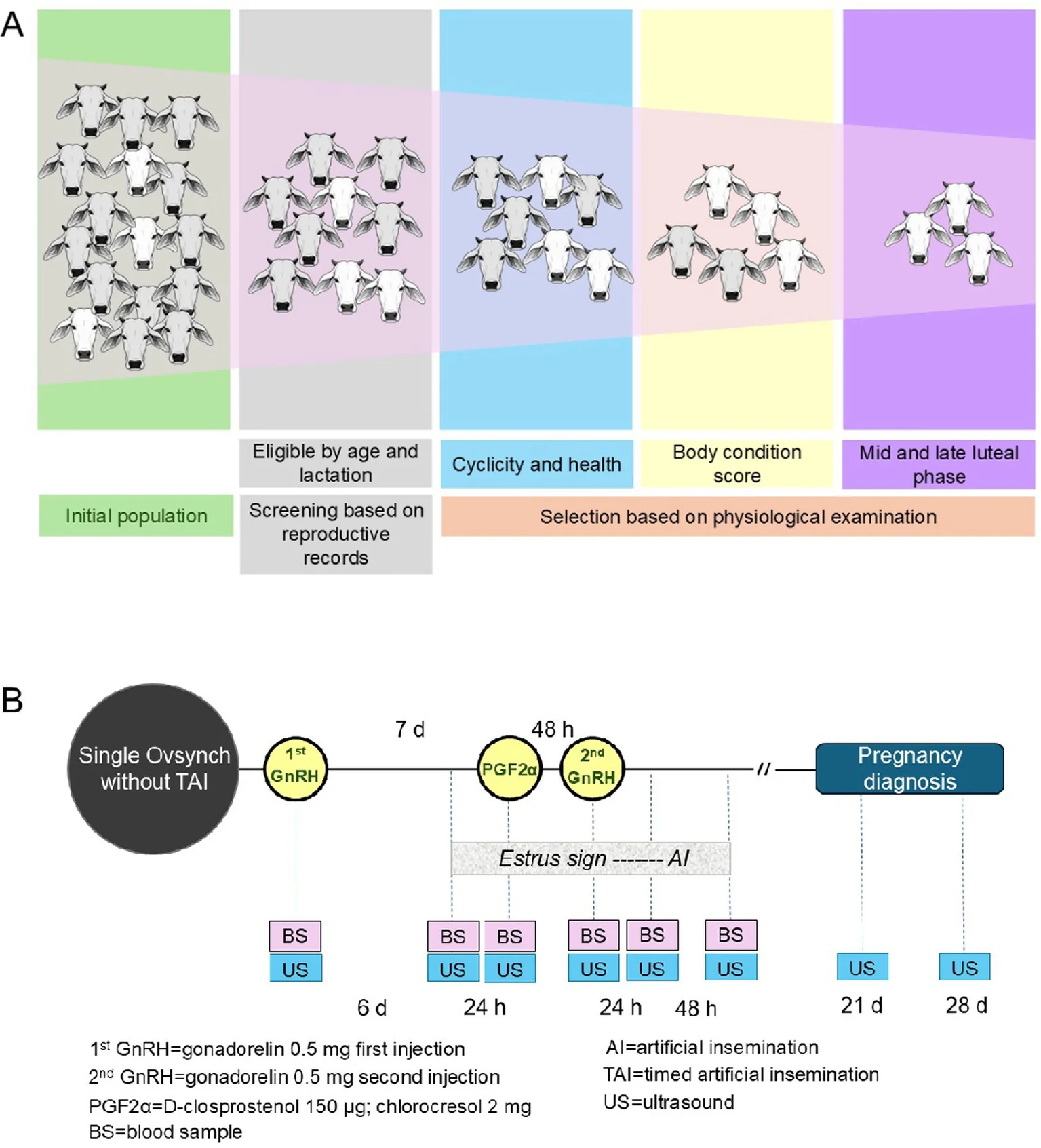
Study design, Ovsynch-based synchronization protocol, artificial insemination and pregnancy diagnosis timeline. **(A)** Sample selection flow, and **(B)** Hormonal protocol adapted from Yamada (13) showing timing of GnRH, PGF2α, AI based on estrus detection, and pregnancy diagnosis.

### 2.3 Modified Ovsynch protocol, artificial insemination and pregnancy diagnosis

The synchronization protocol used in this study followed a GnRH-PGF2α-GnRH sequence adapted from Yamada et al. (13). Cows received a 1st intramuscular injection of GnRH (Fertagyl, 0.5 mg gonadorelin) on Day 0, PGF2α (Indupart, 150 μg D-cloprostenol and 2 mg chlorocresol) on Day 7, followed by a 2nd GnRH injection 48 h later on Day 9. Therefore, the protocol should be interpreted as an Ovsynch-based ovulation synchronization approach with estrus-based insemination, not a conventional fixed-time AI protocol. Estrus was observed every 3 h by inseminators based on standing heat, vulval swelling, and clear cervical mucus. Cows showing estrous signs were then confirmed by a veterinarian before insemination. Artificial insemination was performed using frozen–thawed semen from the same Peranakan Ongole bull, obtained from the Singosari National Artificial Insemination Center (BBIB). Semen from several batches of the same bull was used for insemination following standard thawing procedures. Frozen–thawed semen was routinely evaluated at BPTU and HPT Sembawa according to established quality-control protocol. All AI protocols were performed by the same trained inseminator. Ultrasonographic pregnancy diagnosis was performed by a veterinarian 21 days after AI and reconfirmed on Day 28. Cows with a visible embryonic vesicle at both examinations were classified as pregnant. The timeline of the modified Ovsynch protocol, AI, and pregnancy diagnosis is described in Figure 1B.

### 2.4 Ultrasonographic examination and image analysis

The transrectal ultrasound schedule is depicted in Figure 1B. Transrectal ultrasonography was performed using an 8 MHz linear probe (DRAMINSKI iScan2 Multi RO A7.0–12860S) with gain 7, depth 8 cm, zoom 100%, FPS 18, and focus 2.0 cm. All settings were kept constant throughout the study. Examinations were conducted following blood sampling and prior to hormone administration. All images were stored as BMP files (400 × 480 pixels) for subsequent analysis using ImageJ (National Institutes of Health, United States). Ovarian measurements included the diameters of the largest follicle (LF) and corpus luteum (CL), and corpus hemorrhagicum (CH), calculated as the average of vertical and horizontal measurements. CH echogenicity was quantified in arbitrary units (AU) by placing a standard rectangular area of interest along its contour. To assess intra-observer repeatability of CH echogenicity measurements, five CH images were re-measured three times using the same ROI procedure in ImageJ. The coefficient of variation (CV) was calculated from the repeated measurements. Post-ovulatory variables, including CH diameter, CH echogenicity, and LF diameter, were measured 72 h after the 2nd GnRH injection. Image analysis was performed by an observer blinded to pregnancy outcome, as pregnancy was confirmed only after completion of all ultrasonographic ovarian measurements.

A follicle was considered dominant when it reached a diameter of ≥10 mm and maintained its size while other follicles regressed. The CL was considered functional when its diameter was ≥10 mm, and it exhibited a distinct luteal structure on ultrasonography. Follicular waves were identified when a new LF (>10 mm) emerged following regression of the previous LF.

### 2.5 Hormone profile analysis

Blood samples were collected at each ultrasonographic examination, prior to hormone administration (Figure 1B). Post-ovulatory estradiol and progesterone concentrations were measured 72 h after the 2nd GnRH injection. Blood was collected via jugular venipuncture into tubes containing K3 EDTA and immediately placed on ice until centrifugation. Plasma was separated by centrifugation at 3000 rpm for 10 min at 4 °C and stored at −20 °C until analysis. Plasma progesterone and estradiol concentrations were quantified using commercial ELISA kits from DRG Instruments GmbH (Germany; progesterone: EIA-1561; estradiol: EIA-2693), according to the manufacturer’s instructions. Each assay plate included the manufacturer-provided calibration standards (S0–S6) and Low and High controls. Samples were analyzed in duplicate, and hormone concentrations were calculated from the corresponding standard curve. The analytical performance characteristics reported by the manufacturer included assay sensitivity, recovery, linearity, and intra- and inter-assay precision. According to the manufacturer, assay sensitivities were 0.1 ng/mL for progesterone (intra-assay CV < 8%, inter-assay CV < 10%) and 2.5 pg./mL for estradiol (intra-assay CV < 7%, inter-assay CV < 11%). Absorbance measurements were obtained using a Cavas MP96 spectrophotometer (Monaco) at 450 nm within 10 min of adding the stop solution.

### 2.6 Statistical analysis

Data were analyzed using R version 4.2.0 (R Foundation for Statistical Computing). Given the exploratory nature and limited sample size, all analyses should be interpreted as hypothesis-generating. Continuous data are presented as individual values and descriptive statistics (mean ± SD). Spearman’s rank correlation was used to examine monotonic associations among ovarian and hormonal variables. For exploratory categorical association testing with pregnancy outcome, continuous post-ovulatory variables were dichotomized using pre-specified median-based thresholds. Fisher’s exact test with a one-sided alternative was then applied to evaluate the hypothesized associations between categorical post-ovulatory variables and pregnancy outcome. Cliff’s delta was calculated using the original continuous values to estimate effect sizes. Leave-one-out cross-validation (LOOCV) and permutation testing (10,000 iterations) were used only as internal consistency checks and were not interpreted as external validation of predictive performance. To address the potential loss of information associated with dichotomization, CH diameter was additionally analyzed as a continuous variable using the Mann–Whitney *U* test and receiver operating characteristic (ROC) analysis. The area under the ROC curve (AUC), 95% confidence interval, and Youden-derived threshold were calculated. No definitive inference was based on *p*-values alone. The absence of statistical significance was interpreted as insufficient evidence of association rather than evidence of no biological effect. *p*-values from the exploratory correlation and categorical association analyses were adjusted for multiple comparisons using the Benjamini-Hochberg false discovery rate (FDR) procedure. Statistical significance was considered at an FDR-adjusted *p* < 0.05. A post-hoc sensitivity analysis was performed to determine the minimum standardized effect size detectable with the available sample size of 10 cows, including 4 pregnant and 6 non-pregnant cows, using two-sided α = 0.05 and 80% power to estimate the minimum detectable standardized effect size.

## 3 Results

Individual ovarian and endocrine responses to the Ovsynch protocol varied markedly among cows (Figure 2). Although all animals received the same hormonal treatment, the direction and magnitude of the changes differed between individuals, with some cows exhibiting responses opposite to the expected physiological effect. Changes in estradiol, progesterone, LF diameter, and CL diameter throughout the synchronization protocol are presented in Figure 2.

**Figure 2:**
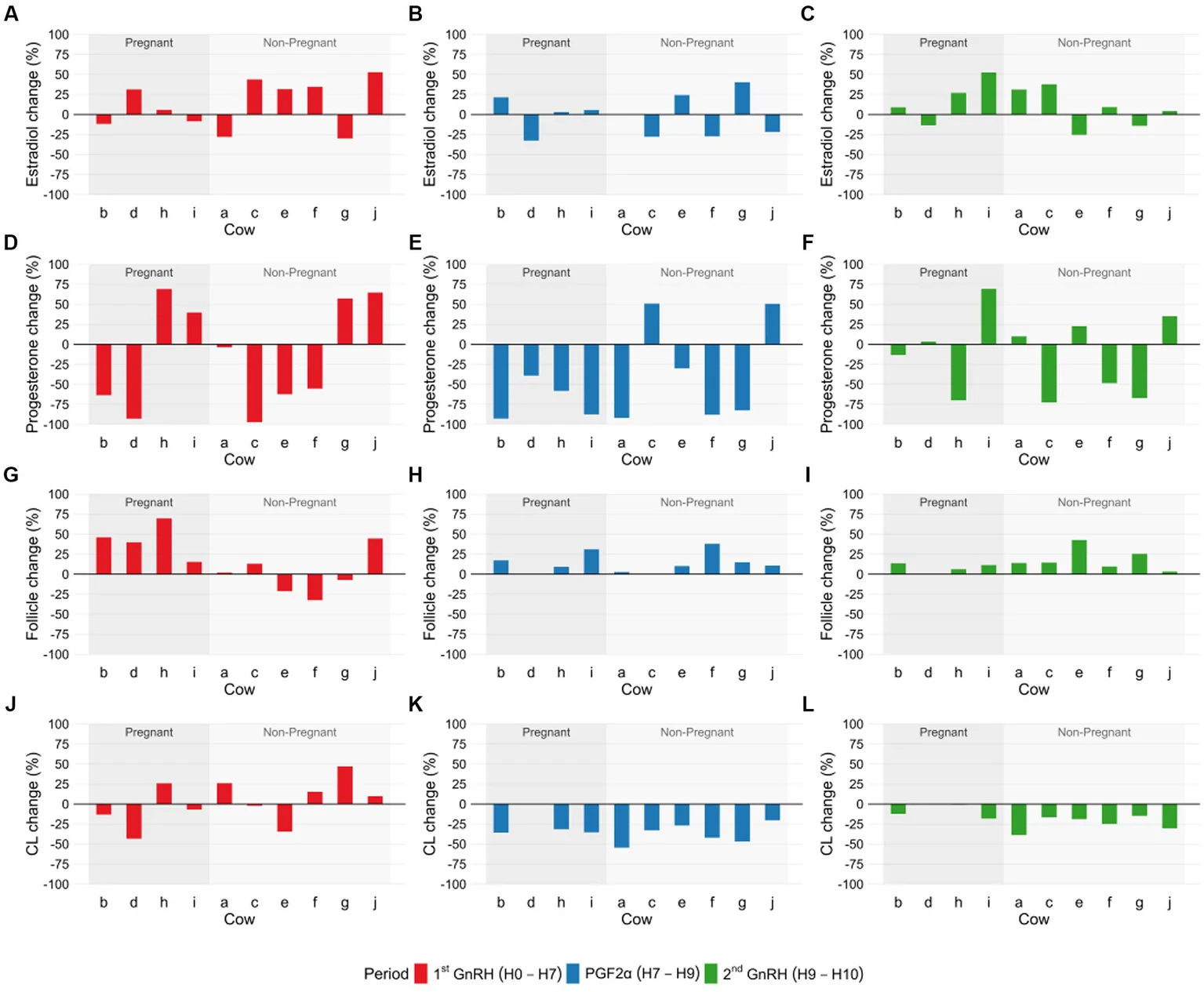
Hormonal and ovarian responses before ovulation during the Ovsynch protocol in individual cows. **(A)** estradiol concentration in cows a-j at 1st GnRH period, **(B)** estradiol concentration in cows a-j at PGF2α period, **(C)** estradiol concentration in cows a-j at 2nd GnRH period, **(D)** progesterone concentration in cows a-j at 1st GnRH period, **(E)** progesterone concentration in cows a-j at PGF2α period, **(F)** progesterone concentration in cows a-j at 2nd GnRH period, **(G)** largest follicle diameter in cows a-j at 1st GnRH period, **(H)** largest follicle diameter in cows a-j at PGF2α period, **(I)** largest follicle diameter in cows a–j at 2nd GnRH period, **(J)** corpus luteum diameter in cows a–j at 1st GnRH period, **(K)** corpus luteum diameter in cows a–j at PGF2α period, **(L)** corpus luteum diameter in cows a–j at 2nd GnRH period.

Changes in estradiol and progesterone concentrations following the 1st GnRH, PGF2α and 2nd GnRH are presented in Figures 2A–F. Following the 1st GnRH injection, estradiol concentrations decreased in 4 cows (a, b, g, i) by 19.51 ± 10.98%, and increased in 6 cows (c, d, e, f, h, j) by 33.26 ± 15.85% (Figure 2A). Following PGF2α administration, estradiol concentrations increased in 6 cows (a, b, e, g, h, i) by 15.78 ± 15.23% and decreased in 4 cows (c, d, f, j) by 27.35 ± 4.61%. Following the 2nd GnRH injection, estradiol concentrations increased in 7 cows (a-c, f, h-j) by 24.39 ± 17.80%, and decreased in 3 cows (d, e, g) by 17.78 ± 6.70%. Following the 1st GnRH injection, progesterone concentrations decreased in 6 cows (a-f) by 62.48 ± 33.57% and increased in 4 cows (g, h, i, j) by 57.64 ± 12.88% (Figure 2D). Following PGF2α administration, progesterone concentrations decreased in 8 cows (a, b, d-i) by 71.26 ± 25.28%, and increased in 2 cows (c, j) by 50.75 ± 0.1%. Following the 2nd GnRH, progesterone concentrations increased in 5 cows (a, d, e, i, j) by 28.11 ± 26.80% and decreased in 5 cows (b, c, f, g, h) by 55.29 ± 25.92%.

Changes in LF and CL diameter throughout the synchronization protocol are presented in Figures 2G–L. Following the 1st GnRH injection, LF diameter increased in 7 cows (a–d, h–j) by 32.87 ± 23.74% and decreased in 3 cows (e, f, g) by 20.33 ± 12.77%. Following PGF2α administration, LF diameter increased in 8 cows (a-c, e-j) by 16.72 ± 11.74%. After the 2nd GnRH injection, LF diameter increased in 9 cows (a–c, e–j) by 15.59 ± 11.89%. Following the 1st GnRH injection, CL diameter increased in 5 cows (a, f, g, h, j) by 24.72 ± 14.25% and decreased in 5 cows (b–e, i) by 19.98 ± 17.97%. Following PGF2α administration, CL diameter decreased in 9 cows (a–c, e–j) by 36.16 ± 10.38%. After the 2nd GnRH injection, CL diameter decreased in 9 cows (a–c, e–j) by 19.52 ± 10.96%.

Ovulation was successfully synchronized in most cows despite differences in follicular wave patterns (Figure 3). Nine of 10 cows (a-c and e-j) ovulated within the expected synchronized window, 24 to 72 h after the 2nd GnRH injection. One cow (d) ovulated earlier, between 7 days after the 1^st^ GnRH injection and 48 h after PGF2α administration. Of the 9 cows that ovulated within the expected window, three were confirmed pregnant at Day 28. Cow d, although ovulating outside the expected window, was also confirmed pregnant, resulting in an overall pregnancy rate of 40% (4/10).

**Figure 3:**
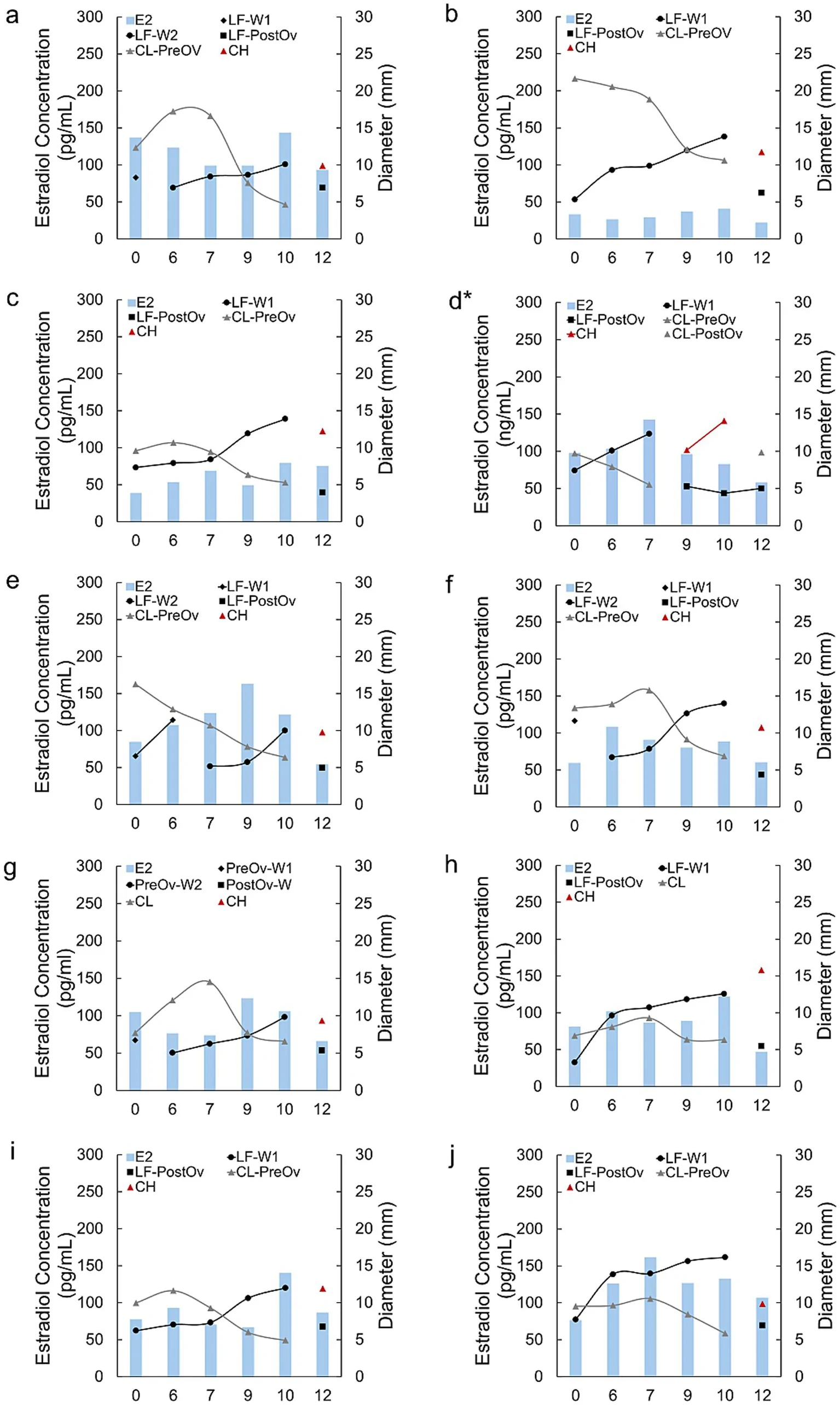
Individual ovarian and follicular wave patterns during the Ovsynch protocol. **a** = cow a, **b** = cow b, **c** = cow c, **d** = cow d, **e** = cow e, **f** = cow f, **g** = cow g, **h** = cow h, **i** = cow i, **j** = cow j. E2 = estradiol, LF-W1 = largest follicle of the first follicular wave, LF-W2 = largest follicle of the second follicular wave, LF-PostOv, largest follicle post-ovulation; CH, corpus hemorrhagicum; CL-PreOv, corpus luteum pre-ovulation, CL-PostOv, corpus luteum post-ovulation. *Cow d exhibited behavioral estrus earlier than the other cows during the synchronization protocol.

Analysis of follicular wave pattern identified six cows (b-d and h-j) with a single-wave pattern, and four cows (a, e, f and g) with double-wave pattern. Single-wave cows had a smaller initial LF diameter (5.91 ± 1.71 mm) but reached a larger pre-ovulatory LF diameter (13.46 ± 1.53 mm) than double-wave cows, which showed an initial LF diameter of 8.30 ± 2.35 mm and a pre-ovulatory LF diameter of 10.98 ± 2.01 mm. Regardless of follicular wave pattern, the pre-ovulatory LF disappeared during the periovulatory period and was subsequently replaced by a corpus hemorrhagicum (CH), confirming successful ovulation. The intra-observer repeatability assessment of CH echogenicity measurements showed a mean CV of 1.57% across the five repeatedly measured CH images.

No significant correlations were observed among post-ovulatory ovarian and endocrine variables measured 72 h after the 2nd GnRH injection (Figure 4). Correlation coefficients ranged from −0.511 to 0.334. The strongest negative correlations were observed between corpus hemorrhagicum characteristics and progesterone (*r* = −0.511 to −0.467), whereas correlations involving estradiol and LF diameter were generally weak (*r* = −0.261 to 0.334).

**Figure 4:**
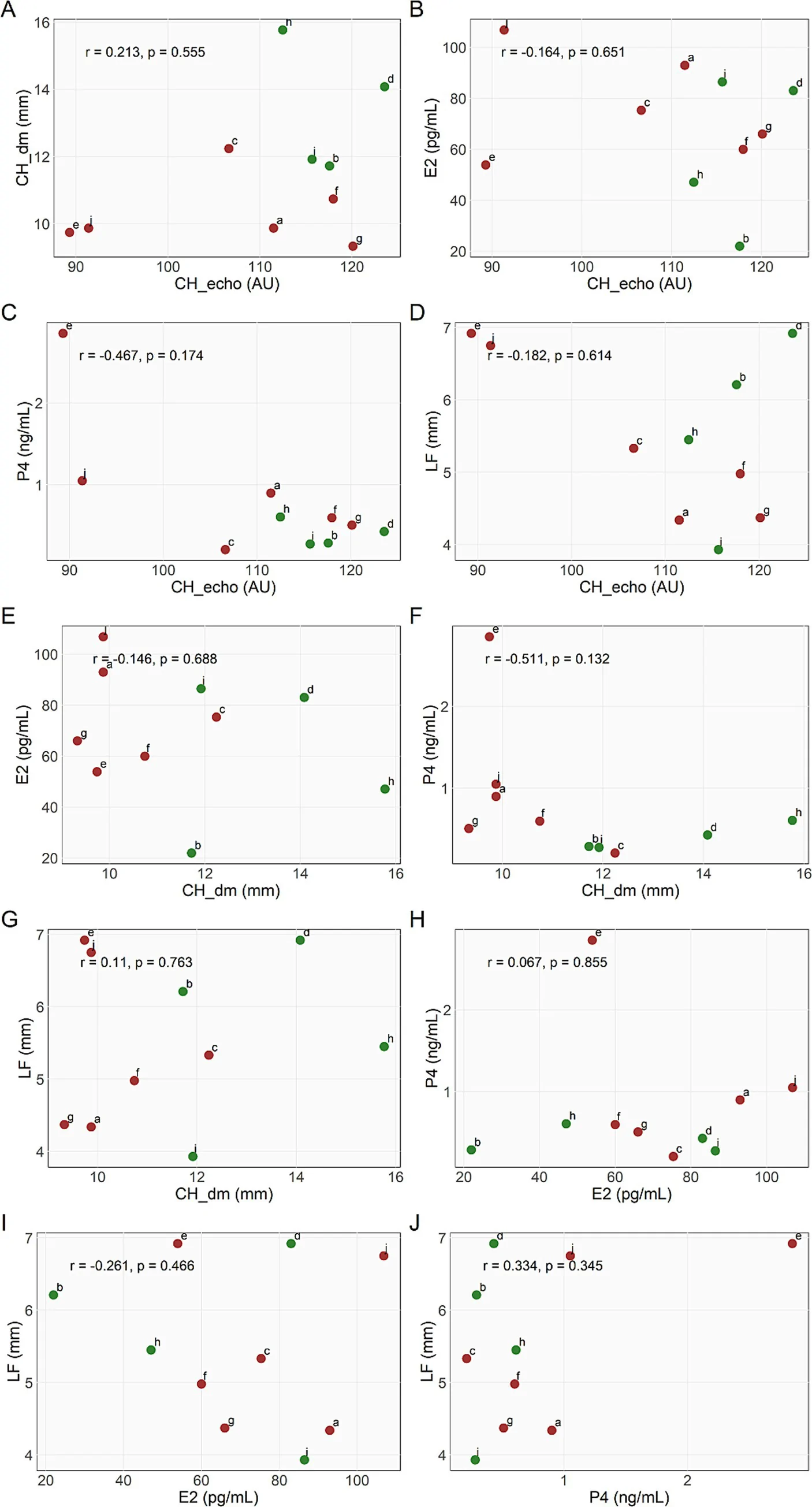
Association among post-ovulatory parameters. **(A)** Association of corpus hemorrhagicum diameter with corpus hemorrhagicum echogenicity, **(B)** Association of estradiol concentration with corpus hemorrhagicum echogenicity, **(C)** Association of progesterone concentration with corpus hemorrhagicum echogenicity, **(D)** Association of largest follicle diameter with corpus hemorrhagicum echogenicity, **(E)** Association of corpus hemorrhagicum diameter with estradiol concentration, **(F)** Association of corpus hemorrhagicum diameter with progesterone concentration, **(G)** Association of corpus hemorrhagicum diameter with largest follicle diameter, **(H)** Association of estradiol concentration with progesterone concentration, **(I)** Association of estradiol concentration with largest follicle diameter, **(J)** Association of progesterone concentration with largest follicle diameter. CH_dm = corpus hemorrhagicum diameter; CH_echo, corpus hemorrhagicum echogenicity; E2, estradiol; P4, progesterone. r, Spearman correlation coefficient.

Baseline ovarian variables were not significantly correlated with post-ovulatory ovarian and hormonal variables (Figure 5). No significant correlations were observed between Day 0 estradiol and post-ovulatory estradiol (*r* = 0.32, *p* = 0.368), Day 0 progesterone and post-ovulatory progesterone (*r* = −0.15, *p* = 0.682), Day 0 LF diameter and post-ovulatory LF diameter (*r* = −0.11, *p* = 0.763), or Day 0 CL diameter and post-ovulatory CH diameter (*r* = −0.22, *p* = 0.532).

**Figure 5:**
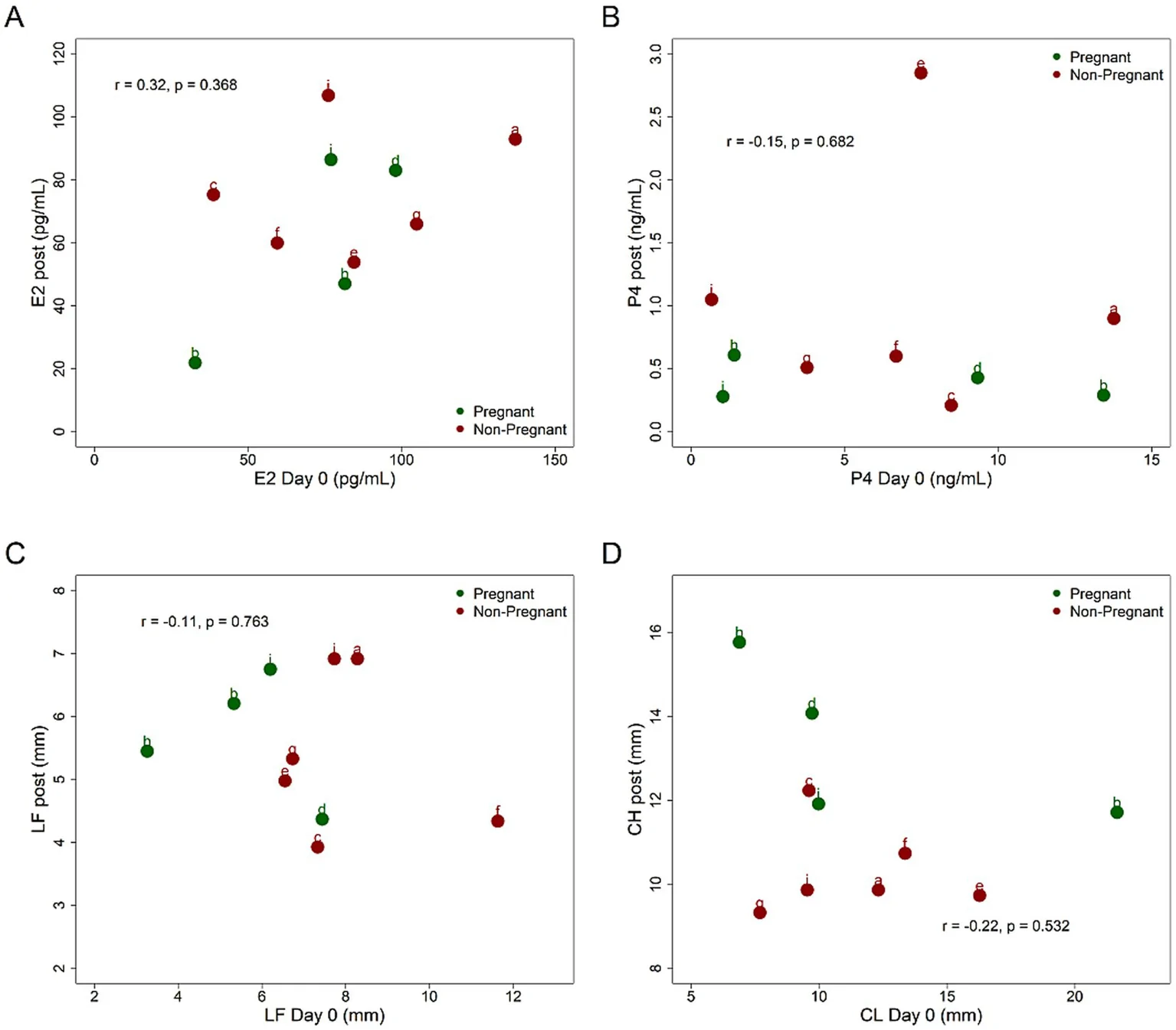
Correlation between Day 0 and post-ovulatory parameters. **(A)** Estradiol, **(B)** Progesterone, **(C)** Largest follicle diameter, **(D)** Corpus luteum diameter versus corpus hemorrhagicum diameter. E2 post = estradiol post-ovulatory; E2 Day 0 = estradiol Day 0; P4 post = progesterone post-ovulatory; P4 Day 0 = progesterone Day 0; LF post = largest follicle post-ovulatory; LF Day 0, largest follicle Day 0; CH post, corpus hemorrhagicum post-ovulatory; CL Day 0, corpus luteum Day 0. *r*, Spearman correlation coefficient.

Pre- and post-ovulatory estradiol concentrations showed a positive correlation, whereas no significant correlations were observed for the other variables (Figure 6). Pre-ovulatory estradiol showed a positive correlation with post-ovulatory estradiol (*r* = 0.68, *p* = 0.035). After FDR correction, this association was not statistically significant (FDR-adjusted *p* = 0.630). In contrast, no significant correlations were observed between pre- and post-ovulatory progesterone concentrations, LF, CL and CH diameter.

**Figure 6:**
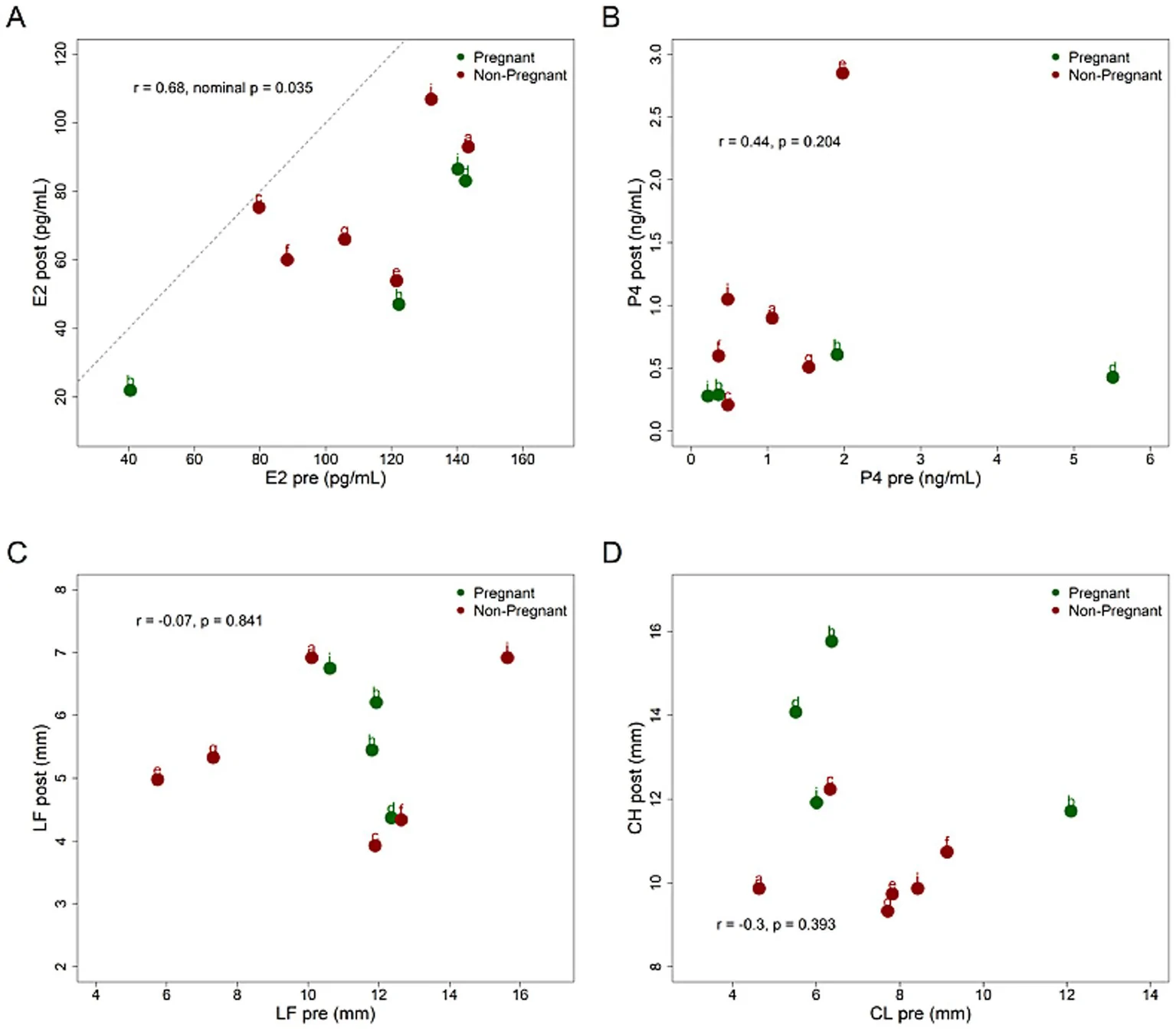
Association between pre-ovulatory and post-ovulatory variables. **(A)** Estradiol, **(B)** Progesterone, **(C)** Largest follicle diameter, **(D)** Corpus luteum diameter and corpus hemorrhagicum diameter. E2 post = post-ovulatory estradiol; E2 pre = pre-ovulatory estradiol; P4 post = post-ovulatory progesterone; P4 pre = pre-ovulatory progesterone; LF post, post-ovulatory largest follicle; LF pre, pre-ovulatory largest follicle; CH post, post-ovulatory corpus hemorrhagicum; CL pre, pre-ovulatory corpus luteum. The dashed diagonal in panel A represents the line of identity (y = x). *r*, Spearman correlation coefficient.

Individual post-ovulatory ovarian profiles are presented in Figure 7. Visual inspection of the normalized data showed that pregnant cows generally exhibited higher CH diameter than non-pregnant cows, whereas the remaining variables showed substantial overlap between groups. Mean normalized CH diameter was higher in pregnant cows than in non-pregnant cows (0.63 versus 0.15). CH echogenicity was also higher in pregnant cows (0.82 versus 0.49), although considerable overlap was observed between groups. In contrast, normalized estradiol (0.44 versus 0.63) and progesterone (0.07 versus 0.31) were lower in pregnant cows, whereas normalized LF diameter was similar between groups.

**Figure 7:**
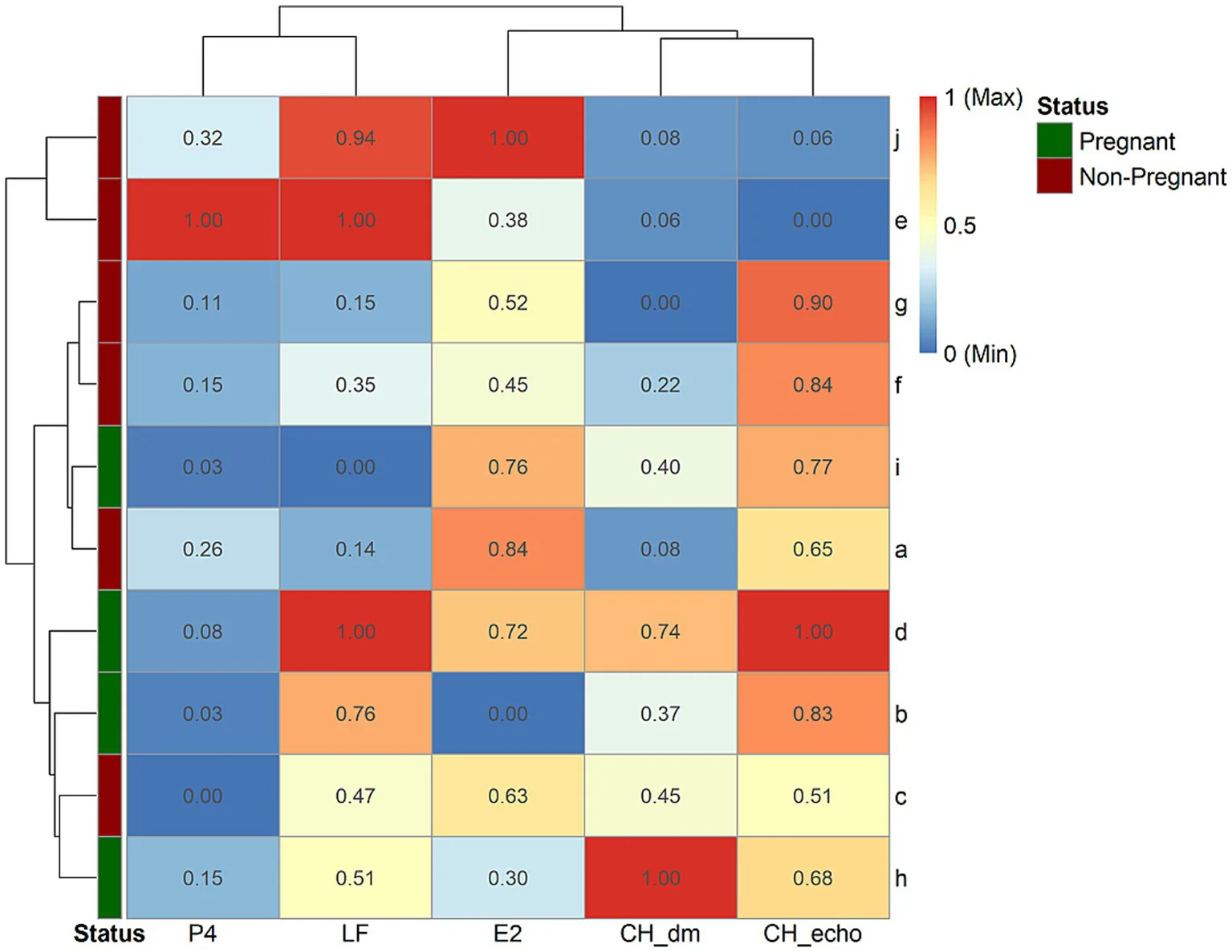
Post-ovulatory profiles for individual cows. Each axis represents normalized values (0–1 scale). a = cow a, b = cow b, c = cow c, d = cow d, e = cow e, f = cow f, g = cow g, h = cow h, i = cow i, j = cow j. E2 = estradiol, P4 = progesterone, CH_echo = corpus hemorrhagicum echogenicity, CH_dm = corpus hemorrhagicum diameter, LF = largest follicle diameter.

Corpus hemorrhagicum (CH) diameter showed the strongest nominal association with pregnancy outcome among all post-ovulatory variables evaluated (Figure 8). The CH diameter category showed a nominal association with pregnancy outcome (Fisher’s exact test, nominal *p* = 0.048), with a large effect size (Cliff’s δ = 0.833). Leave-one-out cross-validation (LOOCV) yielded a classification consistency of 90% and permutation testing yielded *p* = 0.023. After FDR correction, this association was not significant (FDR-adjusted *p* = 0.240). Among the two-variable combinations evaluated, CH diameter combined with LF and CH diameter combined with CH echogenicity also showed nominal associations with pregnancy outcome (Fisher’s exact test, nominal *p* = 0.033 for both combinations). Both combinations showed a classification consistency of 90% in LOOCV. Neither association remained significant after FDR correction (FDR-adjusted *p* = 0.167 for both combinations).

**Figure 8:**
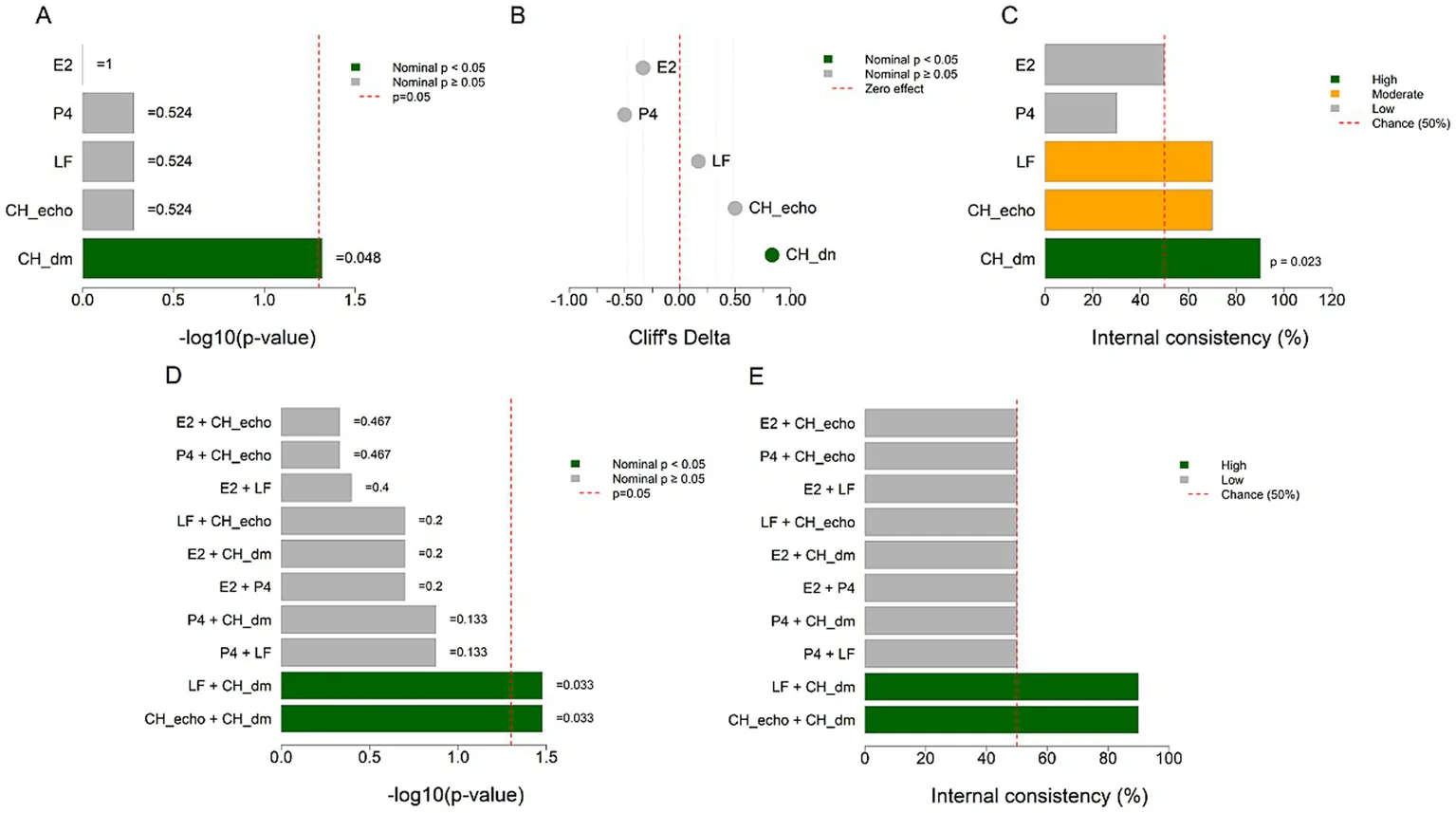
Association between post-ovulatory variables and pregnancy outcome. **(A)** Fisher’s exact test for individual variables, **(B)** Cliff’s delta effect size for individual variables, **(C)** Leave-one-out cross-validation internal consistency for individual variables, **(D)** Fisher’s exact test for two-variable combinations, **(E)** Leave-one-out cross-validation internal consistency for two-variable combinations. CH_dm, corpus hemorrhagicum diameter; CH_echo, corpus hemorrhagicum echogenicity; E2, estradiol; P4, progesterone; LF, largest follicle diameter.

Pregnant cows had a higher median CH diameter of 13.00 mm, ranging from 11.72 to 15.77 mm, compared with 9.87 mm in non-pregnant cows, ranging from 9.33 mm to 12.24 mm (Mann–Whitney *U* test, *p* = 0.042). ROC analysis showed an AUC of 0.917 (95% CI, 0.728–1.000). The Youden-derived threshold was 11.23 mm, with a sensitivity of 100% and specificity of 83.3%. The post-hoc sensitivity analysis indicated that the available sample size of 10 cows, comprising 4 pregnant and 6 non-pregnant, could detect a minimum standardized effect size of approximately Cohen’s d = 2.07 at 80% power.

## 4 Discussion

Although most Peranakan Ongole cows successfully ovulated following the Ovsynch-based synchronization protocol, pregnancy was established in fewer than half of the animals. This finding indicates that successful ovulation alone is insufficient for pregnancy establishment and suggests that events occurring during the periovulatory period may be more closely associated with pregnancy outcome than synchronization success itself. Accordingly, this exploratory longitudinal study examined individual ovarian and hormonal responses throughout the periovulatory period in relation to early pregnancy outcome.

Nine of the ten cows ovulated following the synchronization protocol, suggesting that cows meeting the ultrasonographic selection criteria were generally capable of responding to Ovsynch-based synchronization. Nevertheless, only four cows became pregnant, including one cow that ovulated earlier than expected. This difference between ovulation and pregnancy outcome indicates that successful ovulation did not necessarily result in pregnancy establishment in all cows.

One cow (cow d) ovulated before the 2nd GnRH injection between 7 days after the first GnRH injection and 48 h after PGF2α administration, yet subsequently became pregnant. Early ovulation in this cow may have resulted from a spontaneous endogenous LH surge following PGF2α-induced luteolysis rather than from the exogenous GnRH stimulus. Similar observations have been reported in *Bos indicus* cattle. Barros et al. (14) demonstrated that some Nelore cows ovulated within 48 h after PGF2α administration without requiring a 2^nd^ GnRH-induced LH surge. Likewise, Scarpa et al. (15) reported that approximately 21% of *Bos indicus* crossbred cows ovulated earlier than expected, particularly when the first GnRH injection was omitted. Pfeifer et al. (16) further demonstrated that PGF2α alone can synchronize ovulation in cattle under appropriate concentrations associated with LH release (17). Collectively, these studies suggest that rapid PGF2α-induced luteolysis may occasionally trigger spontaneous ovulation before the scheduled 2nd GnRH injection.

Despite standardized synchronization and uniform ultrasonographic selection, marked inter-individual variation in ovarian responses remained evident. This variability likely reflects differences in the physiological stage of the estrous cycle at protocol initiation that cannot be fully distinguished by B-mode ultrasonography alone. Peranakan Ongole cows exhibit substantial variation in estrous cycle length, both among individuals and across successive cycles, ranging from 14 to 27 days (18). In addition, *Bos indicus* cattle differ in the number of follicular waves occurring during each cycle (19), and Peranakan Ongole cows have been reported to exhibit both three- and four-wave follicular patterns with high repeatability (20). Consequently, variation in follicular wave patterns may contribute to differences in follicular maturity, luteal function, and endocrine status at the onset of synchronization. Such variation has been associated with altered follicular fluid protein composition, inflammatory pathways, oocyte metabolism, and ovulatory processes resulting from differences in progesterone exposure (21).

These physiological differences may help explain why cows responded differently to the same synchronization protocol despite similar ultrasonographic classification at enrollment. One possible explanation is that cows entered the protocol at slightly different stages of luteal development despite meeting the same ultrasonographic selection criteria, which may have influenced their responses to exogenous hormones (22–25). Although more stringent control of the estrus cycle stage at protocol initiation could reduce biological variation, such an approach may also reduce the practical applicability of synchronization protocols under routine field conditions. Nevertheless, ovulation occurred within the expected synchronization window in most cows, indicating that the protocol generally achieved its intended ovulatory response. However, substantial biological variation in the subsequent periovulatory ovarian response remained evident, consistent with previous observations of ovarian dynamics in Peranakan Ongole cattle following Ovsynch-based synchronization (26).

An important consideration is that the present protocol differed from conventional Ovsynch protocols using fixed-time artificial insemination. Insemination was performed following estrus detection and veterinary confirmation, meaning that variation in estrus expression and AI timing may have contributed to variation in pregnancy outcome. In addition, insemination technique may have introduced additional variability. Therefore, pregnancy outcomes observed in the present study should not be directly compared with those reported for conventional fixed-time Ovsynch protocols.

The weak associations observed among post-ovulatory ovarian and hormonal variables may reflect the physiological state of early luteinization at the time of assessment. Following ovulation, the dominant follicle rapidly transforms into a CH, while granulosa and theca cells differentiate into luteal cells and progressively acquire the capacity for progesterone synthesis. During this transition, estradiol production declines as steroidogenesis shifts toward progesterone production (27, 28). However, approximately 72 h after the 2nd GnRH injection, the newly formed CH may not yet have reached full functional maturity. Consequently, structural characteristics such as CH diameter and echogenicity are not necessarily accompanied by proportional changes in circulating progesterone concentrations at this specific time point (27, 29).

This interpretation is supported by studies demonstrating that luteal vascularization and progesterone secretion continue to increase during the first several days after ovulation. Acosta et al. (30) reported progressive increases in luteal blood flow between Day 2 and 5 after ovulation, accompanied by rising circulating progesterone concentrations. Likewise, progesterone secretion remains relatively low during the early stages of luteinization before the CL reaches full functional capacity (28, 31). Therefore, the weak associations observed among post-ovulatory ovarian and hormonal variables may be consistent with the physiological state of early luteinization at the time of assessment.

The absence of detectable associations between baseline ovarian characteristics and post-ovulatory responses suggests that baseline ovarian characteristics were not strongly associated with subsequent post-ovulatory responses. The sequential action of GnRH-induced ovulation or luteinization, followed by PGF2α-induced luteolysis and subsequent follicular recruitment, may reduce the influence of the initial ovarian status on the subsequent periovulatory environment. Consequently, post-ovulatory ovarian characteristics are more likely to reflect the newly established periovulatory response rather than persistence of ovarian conditions present at protocol initiation. This interpretation is supported by previous studies demonstrating comparable pregnancy rates when synchronization protocols were initiated at different luteal stages, provided that ovulation was successfully synchronized (32, 33).

Among the variables evaluated, only pre-ovulatory and post-ovulatory estradiol concentrations showed a positive correlation. This finding suggests that estradiol measured during the early post-ovulatory period may still partially reflect physiological processes associated with the pre-ovulatory follicle.

Following ovulation, estradiol declines as granulosa cells differentiate into luteal cells and progesterone synthesis becomes the dominant steroidogenic pathway (28, 34). However, complete suppression of estradiol production is not immediate, and low circulating estradiol concentrations may persist during the early stages of luteinization (35). Consequently, cows with relatively higher pre-ovulatory estradiol concentrations may continue to exhibit comparatively higher estradiol concentrations shortly after ovulation, which may explain the positive correlation observed in the present study.

Previous studies have shown that declining estradiol concentration following ovulation contributes to the physiological regulation of the reproductive tract by reducing oviductal contractility (36), thereby slowing embryo transport and potentially supporting early embryonic development (37). Because the first post-ovulatory follicular wave has not yet reached the stage of active estradiol secretion (35, 38), circulating estradiol measured 72 h after the 2nd GnRH injection may partly reflect residual estrogenic activity associated with the recently ovulated follicle rather than secretion from newly developing follicles. Although this mechanism cannot be confirmed in the present study, it provides a biologically plausible explanation for why estradiol was the only variable that maintained a detectable correlation across the periovulatory period.

Among the post-ovulatory variables evaluated, CH diameter showed the strongest nominal association with pregnancy outcome. Although this exploratory study included a limited number of animals, pregnant cows exhibited larger CH diameters than non-pregnant cows. This association was supported by a large effect size and high internal consistency during leave-one-out cross-validation.

The corpus hemorrhagicum represents the first structural stage of luteal development immediately after ovulation and serves as the structural precursor of the CL (27, 30, 39). Consequently, variation in CH development during this early period may reflect differences in the progression of luteinization among individual cows. A larger CH may indicate more advanced early luteal development, potentially providing the structural foundation for subsequent CL function. This interpretation is consistent with previous studies reporting positive relationships between luteal size, progesterone production, and pregnancy success later in the luteal phase (29, 40, 41). However, because progesterone concentrations in the present study were measured only during the early post-ovulatory period, it cannot be determined whether the larger CH observed in pregnant cows subsequently developed into a more functional CL.

In addition to CH diameter alone, combining CH diameter with either largest follicle diameter or CH echogenicity showed additional nominal associations with pregnancy outcome. These combinations may provide complementary information because each variable reflects a different aspect of periovulatory ovarian physiology. Whereas the pre-ovulatory follicle reflects follicular development before ovulation, CH diameter represents the earliest structural stages of luteal development after ovulation. Likewise, grayscale CH echogenicity may reflect tissue organization during luteinization rather than luteal vascularization, because Doppler ultrasonography was not performed (42, 43). Therefore, integrating these structural characteristics may provide a more comprehensive assessment of periovulatory ovarian competence than evaluating individual variables alone.

Collectively, these findings suggest that the early post-ovulatory ovarian characteristics may warrant further investigation in relation to pregnancy outcome following synchronization. Despite successful synchronization, the nominal associations observed in this study were primarily related to post-ovulatory characteristics, particularly CH diameter. These observations suggest that evaluation of synchronization success may benefit from considering not only induction of ovulation but also the subsequent periovulatory ovarian response.

Nevertheless, these findings should be interpreted cautiously. The study included only ten cows and was designed as an exploratory investigation. Therefore, the observed associations should be considered hypothesis-generating rather than confirmatory. In addition, AI was performed after detection of estrus rather than at a fixed time, which may have introduced variation in the interval between ovulation and insemination. Although all semen originated from the same bull, semen from several batches was used, and potential batch-to-batch variation could not be separately evaluated in this exploratory study. Other factors that may influence pregnancy outcome, including the uterine environment and early embryonic quality, were not specifically evaluated in this study and therefore could not be controlled for. CH morphology was evaluated using B-mode ultrasonography. Therefore, echogenicity should not be interpreted as a direct indicator of luteal vascularization or blood perfusion (42). An independent in-house assessment of assay parallelism was not performed for the commercial ELISA assays. Therefore, potential matrix-related effects specific to bovine plasma cannot be completely excluded. Furthermore, progesterone concentrations were evaluated only during the early post-ovulatory period and therefore did not characterize subsequent luteal development. Larger, prospectively designed studies incorporating serial luteal assessments, Doppler ultrasonography, and serial hormonal assessments are required to validate these findings and determine whether early post-ovulatory ovarian characteristics can serve as reliable indicators of pregnancy outcome in Peranakan Ongole cows.

## 5 Conclusion

This exploratory longitudinal study examined the relationship between periovulatory ovarian characteristics and pregnancy outcome following Ovsynch synchronization in Peranakan Ongole cows. Corpus hemorrhagicum diameter showed the strongest nominal association among the variables evaluated. These preliminary findings warrant validation in larger prospective studies.

## Data Availability

The original contributions presented in the study are included in the article. Further inquiries can be directed to the corresponding authors.
